# An Exploratory Examination of Health Beliefs and Health Behaviors of Black American Community Dwelling Adults as it Relates to Dementia

**DOI:** 10.1002/alz70858_103993

**Published:** 2025-12-26

**Authors:** Lilcelia A. Williams, Juleen Rodakowski, Monica W Parker

**Affiliations:** ^1^ Department of Occupational Therapy, University of Pittsburgh, School of Health and Rehabilitation Sciences, Bridgeside Point I 100 Technology Drive, PA, USA; ^2^ Department of Medicine Division of Geriatric Medicine, University of Pittsburgh, School of Medicine, Pittsburgh, PA, USA; ^3^ University of Pittsburgh Alzheimer's Disease Research Center (ADRC), Pittsburgh, PA, USA; ^4^ University of Pittsburgh, Pittsburgh, PA, USA; ^5^ Emory University Goizueta Alzheimer's Disease Research Center, Atlanta, GA, USA; ^6^ Emory University, Atlanta, GA, USA

## Abstract

**Background:**

Approximately 7 million people in the United States (US) are living with Alzheimer's disease (AD). Trends from a recent analysis indicates that the incidence of AD in the US is rapidly increasing. Persons who self‐identify as Black Americans have an AD incidence rate twice that of their non‐Hispanic White peers. Further, more older adults die from Alzheimer's disease or a related dementia (ADRD) than breast cancer and prostate cancer combined. Many life threatening and life altering diagnoses such as cancer and diabetes have been studied to examine the relationship between health beliefs and health related behaviors. Yet, few studies have examined the relationship between health beliefs and health related behaviors as it relates to ADRD for persons who self‐identify as Black Americans.

**Method:**

We conducted an exploratory cross‐sectional anonymous survey study to glean insight into our research question; how do health beliefs about dementia influence the health behaviors of community‐dwelling older and aging Black American adults. Persons who self‐identified as Black Americans were purposively sampled from community based events that were hosted by an ADRC located in metropolitan Atlanta, Georgia. Participants (*n* = 34) had a mean age of 69 years (SD±8.38) with 41% (*n* = 14) having a bachelor's degree as their highest level of education. Male participants comprised 59% (*n* = 20) with females accounting for 35% (*n* = 12) and 6% (*n* = 2) did not have a gender indicated. Participants were asked to anonymously complete an online dementia health belief questionnaire which contained questions (*n* = 27) with Likert‐scale responses. Descriptive analyses (plots, frequencies) were generated using IBM SPSS Statistics version 28 to visualize the data.

**Result:**

Our findings illustrated that 38% participants (*n* = 13) agreed to having risk factors while 29% (*n* = 10) agreed to a strong possibility of developing dementia. Of all participants queried, 32% (*n* = 11) were neutral about their chances of developing dementia and 27% (*n* = 9) were neutral about risk factors for developing dementia in the next 10 years.

**Conclusion:**

This exploratory analysis is a first step to gleaning data and forming a deeper understanding about dementia health beliefs and health behaviors within the Black American community. Our findings support the need for additional analysis.

